# Chemical composition of snakes

**DOI:** 10.1371/journal.pone.0266850

**Published:** 2022-06-28

**Authors:** Petra Kölle, Linda F. Böswald, Annita Brenner, Ellen Kienzle

**Affiliations:** 1 Clinic of Small Animal Medicine, Ludwig-Maximilians-Universität München, München, Germany; 2 Chair for Animal Nutrition and Dietetics, Ludwig-Maximilians-Universität München, München, Germany; University of Life Sciences in Lublin, POLAND

## Abstract

The present study was carried out to provide insight into the body composition of snakes, which is an important basis for determination of nutrient requirement and physiological processes. Carcasses of 86 captive snakes (31 pythons, 32 colubrids and 23 boas) were available for analysis. Skins and vertebrae bones of 11 snakes and livers of 64 snakes were analysed separately from the carcasses. Crude nutrients, major minerals and trace elements were investigated. The content of crude nutrients of the whole body was similar to those of mammals and birds. Relatively high contents of copper, zinc and especially of iron (up to 23,973 mg/kg dry matter) were found in the body, particularly in the liver. There was an increase of the iron content of the whole body over age.

## Introduction

Keeping reptiles as pets has become increasingly popular [1, 2]. Still, knowledge on the optimum husbandry, including nutrition, for many reptilian species is limited and often based on experience. In view of animal welfare and disease prevention [3, 4], research on nutrient requirements is important to be able to give diet recommendations. Nutrition-related diseases are well-known [4]. In snakes, this is especially important because the feed animals may not always mirror prey in terms of nutrient supply or if commercial diets are used [5]. Data on body composition is the basis for factorial calculation of nutrient requirements of a species.

Furthermore, whole-body nutrient content is useful for in vivo and post mortem diagnostics. Several parameters of snake body composition have already been investigated [6–12]. Two studies [13, 14] analysed the selenium content of snakes and found high selenium contents especially in animals originating from areas of high pollution. Other investigations target the nutritive value of reptiles for human consumption [e.g. 15].

The objective of this study was to obtain a general overview on the body composition of the different snake groups (boas, pythons, colubrids) and to detect distinctive features of the body composition in comparison to other reptiles, mammals and birds, which may be associated with metabolism and nutrition. In addition, we aimed to identify potential differences in nutrient composition between species and age groups of snakes.

## Material and methods

For the use of dead animals that have not been killed for the experiment, no ethical approval by the government was needed according to German legislation. The Ethical Committee of the Faculty of Veterinary Medicine, LMU München, approved of the study (reference no. 296-11-01-2022). Eighty-six carcasses of snakes of 21 species were analysed. The species belonged to three groups: 31 pythons, 32 colubrids and 23 boas. The animals had died or been euthanized due to various causes and immediately frozen (-18°C) to avoid decomposition of the bodies. The carcasses originated from several pet traders, veterinary practices and private owners, so that specific information about details of the husbandry was not available. Their age varied from hatchling to adult. Hatchlings and snakes not longer than 25% of the expected adult length were defined as juvenile, sexually mature animals were defined as adult and the intermedium stage was specified as semiadult. For measurements and necropsy, the carcasses were thawed over 1–3 days at +4°C. The nutrition status was assessed by the same experienced investigator in all snakes. A descriptive scale from 1 = cachectic, 2 = poor, 3 = moderate, 4 = good, 5 = very good to 6 = obese was used, taking into account species-specific differences in body form. During necropsy, the weights of skins, fat bodies, livers and hearts were recorded. Livers were removed from the carcasses and analysed separately. The gut content was removed from the carcasses. From 11 snakes, skins and 18 vertebrae bones (6 of the cranial proportion, 6 of the medium proportion and 6 of the caudal proportion of the vertebral column) were analysed. Livers of 64 snakes were analysed separately. Results of whole-body including liver, bones and skin were calculated. In 5 skins, selenium content was analysed.

### Chemical analyses

Until analysis, the bodies and organs were stored frozen at -18°C. They were ground (Grindomix GM200^®^, Retsch, Haan, Germany) and lyophilized (Christ Gamma 1–20^®^, Christ, Oderode am Harz, Germany) to determine dry matter (DM). Crude nutrients (crude ash, crude protein, crude fat), minerals (calcium, phosphorus, sodium, potassium, magnesium) and trace elements (copper, zinc and iron) were analysed in skin, vertebrae bones, liver and rest body. Crude nutrients and dry matter were analysed according to Weende analysis [16]. For mineral analysis, wet digestion of samples was carried out in a microwave digestion unit. Calcium, sodium and potassium were analysed by flame emission spectrography [17]. Due to a laboratory error, some calcium data were lost, explaining the lower number of values for calcium than phosphorus. Phosphorus was analysed photometrically with ammonium molybdate and ammonium vanadate in HNO_3_ [18]. Copper, magnesium, zinc and iron were analysed by atomic absorption spectrometry. Selenium was analysed by means of graphite tubing technology.

### Statistical analyses

Statistical comparisons were performed to test for differences between species and age groups. Means and standard deviation (SD) were calculated for each parameter. Means were compared between two groups by student’s t-test or Mann-Whitney Rank Sum Test if normality test failed (p<0.05). If data distribution allowed, One-Way ANOVA was conducted for comparison of more than two means. An all Pairwise Multiple Comparison Procedures depending on distribution of results by Holm-Sidak method or Kruskal-Wallis using Sigmastat^™^ 3.0 as proposed by SigmaStat Pearson correlation coefficient was used in correlation analysis with the exception of assessing correlations regarding nutrition status, which were analysed by Spearman´s rank correlation as a non-parametric measure of statistical dependence.

## Results

### Necropsy

The body weights (BW) ranged from 2.81 to 6476g (mean 441.7g). Proportion of the liver was on average 2.8% of body weight (BW) (range: 1.0–6.0% BW). Skin amounted to 19.1 ± 4.0% BW (mean ± SD). Regarding proportion of liver and skin, there were no statistically significant differences depending on species, age or nutrition status. Proportion of the heart was on average 0.5 ± 0.3% BW (n = 76). The relative weight of the heart showed a correlation with nutrition status (r^2^ = 0.368, p = 0.00114, n = 76). Fat bodies yielded a proportion of 0.00–14.48% BW (on average 3.18% ± 3.72%BW) and showed a strong correlation with BW and nutrition status (r^2^ = - 0.68, p = 0.000, n = 86).

### Chemical analyses

#### Dry matter

Whole-body DM increased with increasing age in pythons and colubrids (Table 1). In boas, whole-body DM showed no statistically significant effect of age. Skin and vertebrae displayed a higher DM content than the whole body (Table 2). Vertebrae bones of three juvenile snakes showed lower DM contents lower than those of adult snakes with 87.5% wet weight (WW) in a juvenile boa and 83.2–84.2%WW in two juvenile pythons.

**Table 1:** Dry matter content (% wet weight) of the whole body in relation to species and age (all values means ± SD; n = number of samples).

Snake group	Juvenile (n)	Semiadult (n)	Adult (n)
Colubrids	24.3 ± 6.2 ^a,AB^ (14)	29.6 ± 6.1^ab,AB^ (2)	31.8 ± 4.0^b,A^ (13)
Pythons	21.0 ± 5.7^a,B^ (14)	26.3 ± 3.8^b,B^ (10)	37.4 ± 4.0 ^c,A^ (4)
Boas	27.7 ± 7.7 ^a,A^ (7)	32.57 ± 2.3 ^a,A^ (4)	31.6 ± 1.8 ^a,A^ (4)

Means in the same line not sharing a superscript small letter differ significantly

Means in the same column not sharing a superscript capital letter differ significantly.

**Table 2:** Dry matter (% wet weight) in different body parts on average of all snakes and in relation to species (all values means ± SD; n = number of samples).

Snake group	Liver (n)	Skin (n)	Vertebrae (n)
Average	22.9 ± 7.6 (68)	36.7 ± 10.7 (11)	89.1 ± 3.1 (11)
Colubrids	27.0 ± 8.5 (24)	36.8 ± 12.6 (3)	89.7 ± 1.9 (3) *
Pythons	20.3 ± 5.9 (28)	30.6 ± 5.7 (3)	90.5 ± 0.0 (1) *
Boas	21.1 ± 6.2 (16)	40.4 ± 12.1 (5)	91.9 ± 0.6 (3) *

*adult specimens.

#### Crude nutrients

The nutrient content in DM of whole body, liver, and bones (see Table 3) did not show statistically significant differences relating to species or age.

**Table 3:** Content of crude nutrients (% DM) in different body parts of snakes (means ± SD; n = number of samples; n.p. = not performed).

Parameter	Whole-body (n)	Liver (n)	Skin (n)	Vertebrae (n)
Crude protein	61.7 ± 8.5 (9)	n.p.	98.4 ± 6.0 (11)	n.p.
Crude fat	16.7 ± 11.9 (28)	16.5 ± 11.9 (21)	1.8 ± 1.5 (11)	0.4 ± 0.6 (11)
Crude ash	17.8 ± 5.7 (25)	4.7 ± 1.0 (21)	3.1 ± 0.8 (2)	63.9 ± 3.7 (11)

There were wide variations in the fat content of the whole body depending on nutrition status in all three snake groups. The whole-body fat content ranged from 2.3% DM in an emaciated juvenile python (Python molurus) to 42.8% DM in an obese adult male corn snake (Pantherophis guttatus). Liver fat content also showed a wide variation (3.4–49.8% DM). There was a significant positive correlation between crude fat content of the liver and crude fat content of the rest of the body (r^2^ = 0.495; p = 0.0266, n = 20).

Whole-body crude ash content increased in average with age (juvenile snakes: 14.6 ± 3.1% DM, n = 6; semiadult snakes: 17.1 ± 5.7% DM, n = 7; adult snakes: 20.9 ± 6.3% DM, n = 9). However, this was not statistically significant due to the small number of samples.

#### Minerals

Calcium content of the whole body varied from 31.24–100.52 g/kg DM (mean 54.73 ± 15.34 g/kg DM; n = 42; see Table 4) and phosphorus content varied from 7.21–70.39 g/kg DM (mean 39.90 ± 11.07 g/kg DM; n = 64; see Table 4).

**Table 4:** Whole-body mineral content of snakes of the three species and age groups (means ± SD; n = number of samples; DM = dry matter).

g/kg DM	Juvenile (n)	Semi-adult (n)	Adult (n)	All ages (n)
Calcium				
• Colubrids	61.7 ± 23.8 ^a,A^ (6)	46.3 ± 20.9 ^a,A^ (2)	67.8 ± 18.0 ^a,A^ (4)	61.2 ± 21.0 (12)
• Phytons	46.0 ± 10.4 ^a,A^ (10)	48.0 ± 17.0 ^a,A^ (5)	48.5 ± 4.0^a,A^ (3)	47.0 ± 11.3 (18)
• Boas	54.5 ± 17.0 ^a,A^ (5)	60.5 ± 18.3 ^a,A^ (3)	64.3 ± 8.6^a,A^ (4)	39.3 ± 29.3 (12)
Phosphorus				
• Colubrids	36.8 ± 6.9 ^a,A^ (6)	42.5 ± 1.2 ^ab,A^ (2)	50.2 ± 12.5 ^b,A^ (13)	45.6 ± 12 (21)
• Phytons	38.7 ± 7.9 ^a,A^ (14)	38.2 ± 5.5 ^a,A^ (10)	31.5 ± 19.0 ^a,A^ (4)	37.5 ± 9.3 (28)
• Boas	39.2 ± 10.8 ^a,A^ (7)	31.7 ± 3.3 ^a,A^ (4)	36.0 ± 14.0 ^a,A^ (4)	36.4 ± 10.3 (15)
Magnesium				
• Colubrids	1.7 ± 0.6 ^a,A^ (6)	1.6 ± 0.3 ^a,A^ (2)	1.9 ± 0.4 ^a,A^ (13)	1.8 ± 0.5 (21)
• Phytons	2.1 ± 0.4 ^a,A^ (14)	1.7 ± 0.2 ^a,A^ (10)	1.3 ± 0.8 ^a,A^ (4)	1.9 ± 0.5 (28)
• Boas	1.9 ± 0.7 ^a,A^ (7)	1.6 ± 0.4 ^a,A^ (4)	1.4 ± 0.4 ^a,A^ (4)	1.7 ± 0.6 (15)
Sodium				
• Colubrids	7.8 ± 5.5 ^a,A^ (5)	5.7 ± 2.3 ^a,A^ (2)	7.5 ± 3.9 ^a,A^ (13)	7.4 ± 3.9 (20)
• Phytons	8.5 ± 4.2 ^a,A^ (14)	10.8 ± 2.0 ^a,B^ (10)	5.1 ± 3.9 ^a,A^ (3)	9.0 ± 3.8 (27)
• Boas	7.1 ± 4.9 ^a,A^ (7)	8.7 ± 1.5 ^a,AB^ (4)	8.0 ± 0.6 ^a,A^ (4)	7.7 ± 3.4 (15)
Potassium				
• Colubrids	7.8 ± 4.0 ^a,A^ (5)	9.2 ± 4.2 ^a,A^ (2)	8.1 ± 2.8 ^a,A^ (13)	8.1 ± 3.1 (20)
• Phytons	11.5 ± 3.9 ^ab,A^ (14)	14.1 ± 4.6 ^a,A^ (10)	6.8 ± 4.8 ^b,A^ (3)	11.9 ± 4.0 (27)
• Boas	9.0 ± 4.0 ^a,A^ (7)	12.6 ± 2.9 ^a,A^ (4)	8.5 ± 1.8 ^a,A^ (4)	9.8 ± 3.5 (15)

Means in the same line not sharing a superscript small letter differ significantly between age groups for one mineral.

Means in the same column not sharing a superscript capital letter differ significantly between species groups for one mineral.

Adult snakes of all groups showed a higher calcium content than juvenile snakes, but this was not statistically significant. Phosphorus content increased with age only in colubrids. The mean whole-body calcium/phosphorus ratio was 1.61 ± 0.96 (n = 42).

There were no differences of whole-body sodium, magnesium and potassium content associated with age or species group. The only exceptions were juvenile and semi-adult pythons showing a statistically significant lower potassium content in the whole body than adult pythons.

For mineral contents of the skin and the vertebrae bones (Table 5), no significant differences with regard to age or species were detected. In the vertebrae bones, the calcium/phosphorus ratio was 2.5 ± 0.4 (n = 11).

**Table 5:** Mineral contents (g/kg DM) in different body parts of snakes (means ± SD; n = number of samples).

Parameter (g/kg DM)	Liver (n = 64)	Skin (n = 11)	Vertebrae (n = 11)
Calcium	1.25 ± 1.26	0.4 ± 0.2	227.5 ± 43.7
Phosphorus	10.2 ± 1.6	2.1 ± 0.8	93.5 ± 22.9
Magnesium	0.63 ± 0.13	0.21 ± 0.09	3.55 ± 0.68
Sodium	9.6 ± 6.2	1.8 ± 0.8	17.5 ± 21.8
Potassium	13.3 ± 5.4	2.7 ± 1.0	13.8 ± 28.9

#### Trace elements

Whole body copper and zinc contents (Table 6) did not show statistically significant differences between age or species groups.

**Table 6:** Copper and zinc contents (mg/kg DM) in different body parts of snakes (means ± SD; n = number of samples).

Parameter (mg/kg DM)	Whole-body (n = 64)	Liver (n = 64)	Skin (n = 11)	Vertebrae (n = 11)
Copper	26.7 ± 29.9	28.0 ± 29.7	1.1 ± 0.6	15.5 ± 8.7
Zinc	255 ± 74	115 ± 41	35 ± 9	131 ± 91

**Fig 1:**
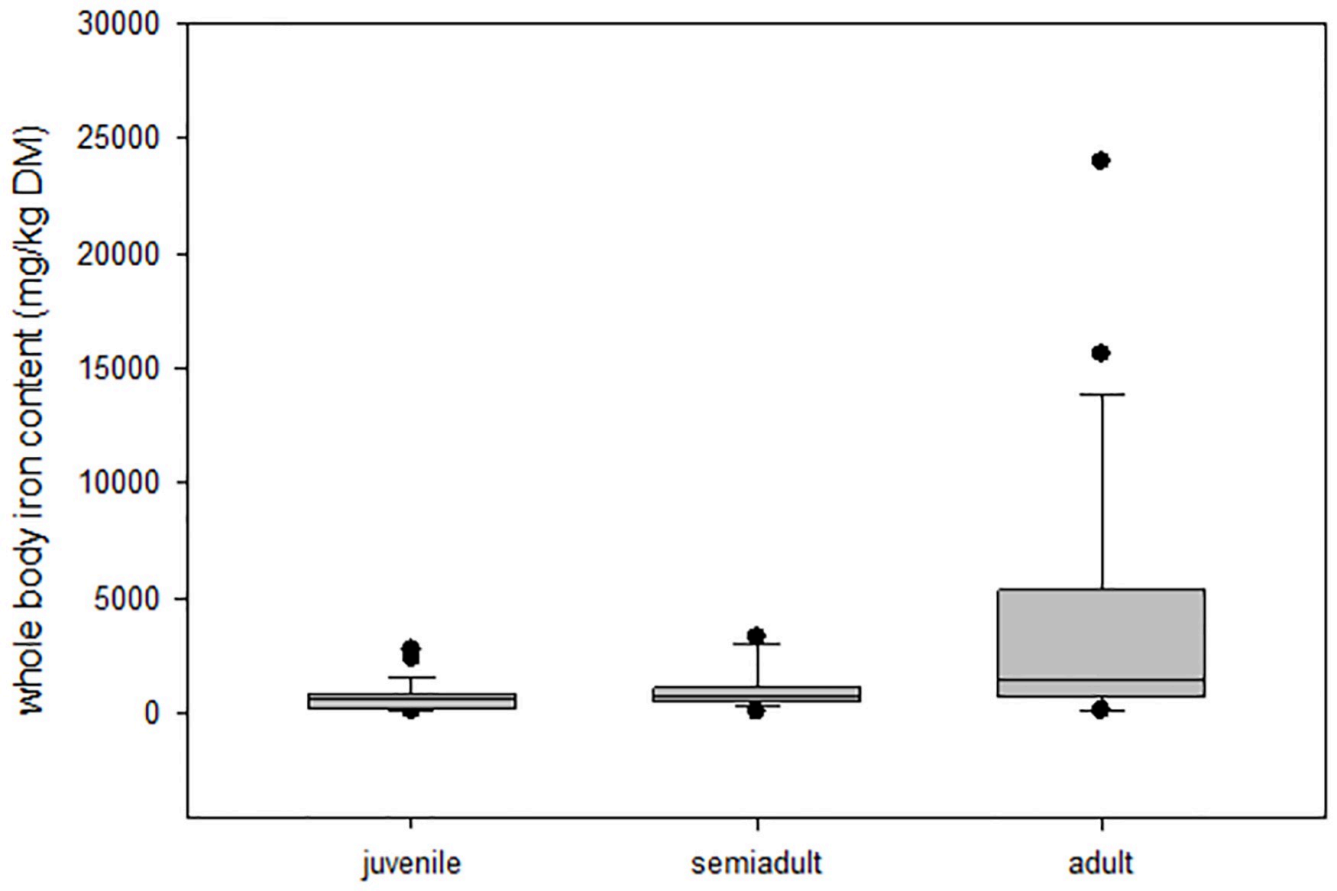
Iron content (mg/kg DM) of the whole body of juvenile, semi-adult and adult snakes (line in the box indicates the median, boxes represent lower quartile to upper quartile (25–75%), lower whiskers 25% − 1.5 ∙ interquartile range, upper whisker: 75% + 1.5 ∙ interquartile Range, dots indicate outliers).

Iron content in the whole body (Table 7) and in the liver was strongly varying (whole body: 103–23,973 mg/kg DM; liver: 95–23,870 mg/kg DM) and increased in colubrids, pythons and boas with increasing age. However, due to the small sample size per group, this trend was not statistically significant within species limits. There was no correlation between the iron content of the rest body and the iron content of the liver (r^2^ = 0.181, p = 0.2, n = 52) and no correlation between iron content of the whole body and the nutrition status (r^2^ = 0.136, p = 0.337, n = 52). If all juvenile, semiadult and adult snakes are regarded, there is a statistically significant increase of iron content of the whole body with age (juvenile vs. adult, p<0.05; Fig 1).

**Table 7:** Whole-body iron content (mg/kg DM) in the age and species groups of snakes (means ± SD; n = number of samples).

Snake group	Juvenile (n)	Semiadult (n)	Adult (n)
Colubrids	508 ± 483 (6)	410 ± 433.7 (2)	5003 ± 7120 (13)
Pythons	831 ± 675 (14)	1191 ± 1062 (10)	3069 ± 2786 (4)
Boas	757 ± 775 (7)	1140 ± 343 (4)	1261 ± 992 (4)

In skin (29 ± 11 mg/kg DM; n = 11) and vertebrae bones (28 ± 12 mg/kg DM; n = 11), only small amounts of iron were detected.

In the body without liver, selenium content was 174.1 μg/kg DM (range: 99.5–352.8 μg/kg DM; n = 10). Selenium content of the skin was on average 94.8 ± 96.3 μg/kg wet weight (range: 28.8–255.5 μg/kg wet weight; n = 5) and 254.1 ± 275.8 μg/kg DM (87.7–737.8 μg/kg DM; n = 5).

## Discussion

The results of the present study should be interpreted cautiously because material originated not from healthy specimens but from animals, that died or were euthanized due to different causes, including acute infections or trauma. Because of the lack of history information, no separation of data according to disease(s) could be performed. The snakes also showed a variety of body condition/nutrition status from cachectic to obese. With the exception of fat content in whole body and liver, however, correlations between nutrition status and other tested parameters showed to be low or absent. Snakes are adapted to periods of starvation and can regulate to a certain degree which body parts will be used for energy mobilisation first [19]. This might have influenced the direct relationship between nutrition status and body fat storage.

Analysis was performed after frozen storage for varying time spans. The nutrients investigated are stable at -18°C, so that no bias due to storage of the carcasses is expected.

### Necropsy

Proportion of the liver of the body weight was similar to that in mammals and birds [20–23]. Liver weight in proportion of body weight of hatchlings was smaller than in neonate mammals. In contrast to mammal neonates, there is no storage of glycogen in the liver, as reptilian hatchlings possess a yolk sac for energy supply during the first days after hatching. There is generally a high variation of liver weight in reptiles, depending on nutrition status, reproduction period, and season [10, 24].

As expected, there was a strong correlation between weight of fat bodies and nutrition status. This is because in snakes, storage of fat occurs beside storage in the liver nearly exclusively in fat bodies located caudal in the coelom. Only in some obese snakes, small fat bodies around the myocardium can be found, but there is no subcutaneous fat tissue in reptiles like it is found in mammals.

Skin proportion of body weight in this study was similar to results in literature [25]. Proportions of the skin of boas, pythons and colubrids were similar, although it was assumed that there should be bigger variations in the groups due to different body shape and different surface/volume ratio of boas and pythons compared to colubrids due to their larger cross section dimension.

### Chemical analyses

Results of DM and nutrients are comparable to literature data (see Table 8) on body composition of different species of reptiles, birds and mammals [21, 26–34]. Even though there are differences in the natural feeding habits of the species listed, the overall dimension of nutrient content is similar. The snakes of our study did not show a systematic deviation from literature data.

**Table 8:** Comparison of the whole-body DM and nutrient content of different reptilian, avian and mammalian species (n = number of observations).

Species (adult specimens, if not cited otherwise)	DM (% WW) (n)	Crude fat (% DM) (n)	Crude protein (%DM) (n)	Crude ash (% DM) (n)	Literature
Snakes (boas, pythons and colubrids)	27.4 (72)	16.7 (28)	61.7 (9)	17.8 (25)	This study
Eastern Fence Lizard (*Sceloporus undulatus*)	23.4 (37)	18.4 (37)	-	-	Angiletta (1992)
Anole (*Anolis carolinenesis*)	29.4 (19)	-	67.4 (13)	-	Cosgrove et al. (2002)
Bearded Dragon (*Pogona vitticeps*)	17.9 (17)	-	63.7 (17)	-	Cosgrove et al. (2002)
Chicken (juvenile) (*Gallus gallus domesticus*)	22.8 (11)	16.5 (11)	67.7 (11)	8.2 (11)	Dierenfeld et al. (2002)
Chicken (adult) (*Gallus gallus domesticus*)	40.5 (1)	51.1 (1)	45.0 (1)	6.2 (1)	Dierenfeld et al. (2002)
Cockatiel (*Nymphicus hollandicus*)	39.0 (9)	11.2 (9)	70.4 (9)	12.4 (9)	Rabehl (1995)
Hamster (*Mesocricetus auratus*)	30.0 (n?)	35.0 (n?)	50.0 (n?)	8.0 (n?)	Tabaka et al. (1996)
Mouse (*Mus musculus*)	32.6 (7)	23.5 (7)	56.9 (7)	11.3 (7)	Dierenfeld et al. (2002)
Rat (*Rattus norvegicus var*. *dom*.)	30.2 (5)	24.0 (5)	60.1 (5)	15.9 (5)	Douglas et al. (1994)
Dog (*Canis lupus familiaris*)	43.9 (53)	51.1 (53)	36.7 (53)	8.1 (53)	Stadtfeld (1978)

As limbs are lacking in snakes, the amount of bone and consequently the whole-body calcium content was expected to be lower compared to mammals, but no such effect was found. Calcium content of snakes was even higher than in most mammals (see Table 9) and the calcium/phosphorus ratio in snakes (mean 1.61) is similar as in mammals. This is in accordance with literature data [8, 35, 36]. A study on tissue composition of Wagler snakes, *Xenodon merremii wagler*, [37] found highest calcium content in bones, followed by brain, glottis, eggs and trachea and the lowest concentrations in muscles and organs. They report extremely high calcium/phosphorus ratios of 4.4 in snake maxilla and toad humerus and femur. In the present study, vertebrae had a calcium/phosphorus ratio of 2.4/1, which is comparable to mammalian bones.

**Table 9:** Comparison of whole-body mineral content of different reptilian, avian and mammalian species.

Species*	n	Calcium (g/kg DM)	Phosphorus (g/kg DM)	Sodium (g/kg DM)	Potassium (g/kg DM)	Magnesium (g/kg DM)	Literature
Snakes	43–64	54.61	39.9	15.2	23.7	1.8	This study
Bearded Dragon (*Pogona vitticeps*)	6	34.2	23.6	7.0	12.0	1.5	Cosgrove et al (2002)
Cockatiel (*Nymphicus hollandicus*)	9	42.9	19.8	3.3	6.2	1.1	Rabehl (1995)
Mouse (*Mus musculus*)	7	26.4	19.1	4.3	10.2	1.3	Dierenfeld et al (2002)
Rat (*Rattus norvegicus var*. *dom*.)	22	34.5	19.1	4.3	10.5	1.5	Dierenfeld et al (2002)
Dog (*Canis lupus familiaris*)	53	24.1	13.1	2.8	3.9	0.6	Stadtfeld (1978)

*adult species, if not cited otherwise.

Whole-body mineral contents were on average higher than in mammals and birds (see Table 9).

On average, the snakes investigated in this study had higher whole-body iron, zinc and copper contents than birds and mammals from literature data (see Table 10).

**Table 10:** Comparison of whole-body iron, copper and zinc content of different reptilian, avian and mammalian species.

Species*	n	Iron (mg/kg DM)	Copper (mg/kg DM)	Zinc (mg/kg DM)	Literature
Colubrids	13	5,003	44.6	282	This study
Pythons	4	1,261	13.0	217	This study
Boas	4	3,068	41.3	252	This study
Brown Water Snake (*Nerodia taxispilota*)	10	1,779	3.0	151	Burger et al. (2006)
Bearded dragon (*Pogona vitticeps*) juvenile	5	145	11.3	155	Cosgrove et al. (2002)
Chinese alligator (*Alligator sinensis*)	2	67	6.4	125	Xu et al. (2006)
Chicken (*Gallus gallus domesticus)*, juvenile	11	157	4.0	94	Dierenfeld et al. (2002)
Cockatiel (*Nymphicus hollandicus*)	9	199	20.7	141	Rabehl (1995)
Mouse (*Mus musculus*)	7	251	8.0	89	Dierenfeld et al. (2002)
Rat (*Rattus norvegicus var*. *dom*.)	22	195	7.5	92	Dierenfeld et al. (2002)

*adult species, if not cited otherwise.

As the size of the liver samples was limited, only selenium content of the rest body and skin could be analysed. Results were comparable to those described for the water snake *Nerodia taxispilota* (200 μg/kg DM) [34]. However, in pine snakes (*Pituophis melanoleucus)* [34], sea turtles (*Caretta caretta*) [38] and alligators (*Alligator mississippiensis*) [39], much higher selenium contents up to 119,000 μg/kg WW were found. Differentiating by tissue, on study [40] found highest selenium concentrations in kidney and liver of pine snakes. Reptiles seem to have a high tolerance for selenium supply and selenium storage is probably an indicator of environmental selenium concentration of natural origin or pollution. As predators, snakes will accumulate such pollutants they take up through prey [40]. In our studies, the analysed snakes were mainly bred in captivity, so pollution might not be a major contributor to selenium content.

The snakes in this study showed a remarkably high whole-body iron content. It is unclear whether such high iron contents are physiological or pathological. Since the snakes are predators, the majority eating small vertebrates as their natural diet, it is unlikely that their diet in captivity would contain more iron than their diet in the wild. Therefore, decreased iron tolerance can be excluded as a major cause for the observed high whole-body iron levels.

Whole-body iron content increased with age, but even the juvenile individuals had high values. For chelonians, high iron contents have been reported as well [41]. These findings may indicate that the high amount of iron in the snake bodies are rather physiological or at least do not have negative effects on development and adult reproduction. Free-ranging snakes have a much lower life expectancy than captive snakes. If iron accumulates in the body with age, they might not reach an age where high iron concentrations cause harm. In this case, it would not be necessary for snakes to have evolved a protective mechanism against high iron intakes, compared to other species [42, 43].

Another possible explanation for the high whole-body iron concentrations may be the reaction to an inflammatory state, causing a change in trace element distribution throughout the body [44, 45]. Iron levels may influence the immune response.

In contrast to the present results with increasing values from juvenile to adult snakes, a different study [27] found whole-body iron contents decreasing with age in bearded dragons (*Pogona vitteceps*). The change of dietary habits in lizards is suggested as a reason for the decrease in iron content. Snakes, however, do not change from carnivorous to herbivorous throughout their development, thus accumulation of iron from prey may be a likely reason for the increasing values. A study [37] found highest iron concentrations in the liver and spleen of Wagler snakes (*Xenodon merremii wagler)*. This correspondents with high iron concentrations in the livers of chelonians [41].

Copper has been reported to accumulate in bone, liver and brain of snakes [37]. The mean liver copper content of the snakes analysed in this study is far below values for different mammalian species (e.g. bovine 470mg/kg DM, sheep up to 599 mg/kg DM, equine 219–349 mg/kg DM) [46]. Snakes do not seem to accumulate and store copper in the liver in amounts like ruminants.

The concentrations of copper and zinc in the skin is in accordance to literature stating the lowest trace element concentrations in snake bodies in the skin [47].

It needs to be taken into account that the present study provides data on captive snakes, while most literature sources have investigated free-ranging animals (road-kill, traps etc.). There may be differences in body composition due to dietary nutrient supply from anthropogenic diets vs. wild prey animals, which also underlie seasonal and habitat variation [48].

## References

[1] Moriarty J.J., Reptiles as pets: an examination of the trade in live reptiles in the United States. Herpetological Review, 2002. 33(3): p. 236.

[2] Burghardt G., Keeping reptiles and amphibians as pets: challenges and rewards. Vet. Rec, 2017. 181: p. 447–449. doi: 10.1136/vr.j4912 29074794

[3] Donoghue S. and Langenberg J., Clinical nutrition of exotic pets. Australian Veterinary Journal, 1994. 71(10): p. 337–341. doi: 10.1111/j.1751-0813.1994.tb00915.x 7848183

[4] Pasmans F., et al., Future of keeping pet reptiles and amphibians: towards integrating animal welfare, human health and environmental sustainability. Veterinary Record, 2017. 181(17): p. 450–450. doi: 10.1136/vr.104296 29051315

[5] Kölle, P., *Echsen und Schlangen*: *Heimtier und Patient*. 2015: Georg Thieme Verlag.

[6] Benedict, F.G., *The physiology of large reptiles*: *with special reference to the heat production of snakes*, *tortoises*, *lizards and alligators*. 1932.

[7] Khalil F. and Abdel-Messeih G., Water content of tissues of some desert reptiles and mammals. Journal of Experimental Zoology, 1954. 125(3): p. 407–414.

[8] Jenkins N. and Simkiss K., The calcium and phosphate metabolism of reproducing reptiles with particular reference to the adder (Vipera berus). Comparative Biochemistry and Physiology, 1968. 26(3): p. 865–876. doi: 10.1016/0010-406x(68)90006-6 5758313

[9] Thorson T.B., Body fluid partitioning in Reptilia. Copeia, 1968: p. 592–601.

[10] Aleksiuk M. and Stewart K.W., Seasonal changes in the body composition of the garter snake (Thamnophis sirtalis parietalis) at northern lattitudes. Ecology, 1971. 52(3): p. 485–490.

[11] Vitt L.J., Caloric content of lizard and snake (Reptilia) eggs and bodies and the conversion of weight to caloric data. Journal of Herpetology, 1978: p. 65–72.

[12] Secor S.M. and Nagy T.R., Non-invasive measure of body composition of snakes using dual-energy x-ray absorptiometry. Comparative Biochemistry and Physiology Part A: Molecular & Integrative Physiology, 2003. 136(2): p. 379–389. doi: 10.1016/s1095-6433(03)00176-4 14511756

[13] Burger J., Trace element levels in pine snake hatchlings: tissue and temporal differences. Archives of Environmental Contamination and Toxicology, 1992. 22(2): p. 209–213. doi: 10.1007/BF00213287 1536601

[14] Hopkins W.A., Rowe C.L., and Congdon J.D., Elevated trace element concentrations and standard metabolic rate in banded water snakes (Nerodia fasciata) exposed to coal combustion wastes. Environmental Toxicology and Chemistry: An International Journal, 1999. 18(6): p. 1258–1263.

[15] Wang D., Tang Z., and Tan Y., Biochemical compositions of Chinese soft-shelled turtle (Trionyx sinensis) I. Contents of normal nutrients and composition of muscle fatty acids. Acta Hydrobiologica Sinica, 1997. 21: p. 305–310.

[16] Naumann, C. and R. Bassler, *Die chemische Untersuchung von Futtermitteln*. *Band III*: *Methodenbuch*. 1986, Neudamm, Germany: Verlag J. Naumann.

[17] Schuhknecht W., Universalvorschrift fur die Bestimmung von Kalium, Natrium und Lithium nebeneinander. Z. Analyt. Chem., 1963. 194: p. 176–183.

[18] Gericke S. and Kurmies B., Colorimetrische Bestimmung der Phosphorsäure mit Vanadat-Molybdat. Fresenius’ Zeitschrift für analytische Chemie, 1952. 137(1): p. 15–22.

[19] McCue M.D., Lillywhite H.B., and Beaupre S.J., *Physiological responses to starvation in snakes*: *low energy specialists*, *in Comparative physiology of fasting*, *starvation*, *and food limitation*. 2012, Springer. p. 103–131.

[20] Meyer, H. and C. Dammers, *Untersuchungen zum Energie-und Nährstoffbedarf von Zuchthündinnen und Saugwelpen*. 1985: P. Parey.

[21] Rabehl, N., *Untersuchungen zur Körperzusammensetzung und deren Entwicklung bei verschiedenen Ziervogelarten (Kanarien*, *Wellensittiche*, *Agaporniden*, *Nymphensittiche*, *Amazonen und Graupapageien)*. 1995.

[22] Stratmann, B., *Untersuchungen zur Körperzusammensetzung von Katzen (Investigations on body composition of cats)*. 1988, Doctoral Thesis, Hannover: Tierärztliche Hochschule.

[23] Rosenberg von, S., *Untersuchungen zur knochenprtektiven Wirkung von Trisetum flavescens und Solanum malacoxylon im Osteoporosemodell von der ovariektomierten Ratte*., *in Tierärztliche Fakultät*. 2005, Ludwig-Maximilians-Universität Müchen.

[24] Santos X. and Llorente G. A., Lipid dynamics in the viperine snake, Natrix maura, from the Ebro Delta (NE Spain). Oikos, 2004. 105(1): p. 132–140.

[25] Bonnet X. and Naulleau G., Are body reserves important for reproduction in male dark green snakes (Colubridae: Coluber viridiflavus)? Herpetologica, 1996: p. 137–146.

[26] Angilletta M.J. Jr, Estimating body composition of lizards from total body electrical conductivity and total body water. Copeia, 1999: p. 587–595.

[27] Cosgrove J.J., et al., Whole-body nutrient composition of various ages of captive-bred bearded dragons (Pogona vitteceps) and adult wild anoles (Anolis carolinensis). Zoo Biology: Published in affiliation with the American Zoo and Aquarium Association, 2002. 21(5): p. 489–497.

[28] Dierenfeld, E.S., H.L. Alcorn, and K.L. Jacobsen, *Nutrient composition of whole vertebrate prey (excluding fish) fed in zoos*. 2002: US Department of Agriculture, Agricultural Research Service.

[29] Douglas T.C., Pennine M., and Dierenfeld E.S., Vitamins E and A, and proximate composition of whole mice and rats used as feed. Comparative Biochemistry and Physiology Part A: Physiology, 1994. 107(2): p. 419–424. doi: 10.1016/0300-9629(94)90401-4 7907967

[30] Stadfeld, G., *Untersuchungen über die Körperzusammensetzung des Hundes*., in *Tierärztliche Fakultät*. 1978, Ludwig-Maximilians-Universität München.

[31] Tabaka C.S., et al., Diet, cast composition, and energy and nutrient intake of red-tailed hawks (Buteo jamaicensis), great horned owls (Bubo virginianus), and turkey vultures (Cathartes aura). Journal of Zoo and Wildlife Medicine, 1996: p. 187–196.

[32] Stadtfeld, G.n., *Studies on body composition of dog*. 1978, Tierärztliche Hochschule Hannover: Hanover, Germany.

[33] Xu Q., et al., Heavy metal distribution in tissues and eggs of Chinese alligator (Alligator sinensis). Archives of environmental contamination and toxicology, 2006. 50(4): p. 580–586. doi: 10.1007/s00244-005-1018-3 16489418

[34] Burger J., et al., Element levels in snakes in South Carolina: differences between a control site and exposed site on the Savannah River site. Environmental Monitoring and Assessment, 2006. 112(1): p. 35–52. doi: 10.1007/s10661-006-0695-3 16404533

[35] Anderson J.F., Rahn H., and Prange H.D., Scaling of supportive tissue mass. The quarterly review of biology, 1979. 54(2): p. 139–148.

[36] Packard M.J. and Packard G.C., Sources of calcium and phosphorus during embryogenesis in bullsnakes (Pituophis melanoleucus). Journal of Experimental Zoology, 1988. 246(2): p. 132–138.

[37] De Jorge F., Do Amaral A., and Abe A., Biochemical studies on the snake, Xenodon merremii wagler, 1824 (reptilia, ophidia, colubridae) as compared with the toad, Bufo marinus ictericus spix, 1824 (Amphibia, salientia, bufonidae). Comparative Biochemistry and Physiology Part B: Comparative Biochemistry, 1971. 38(3): p. 553–583.

[38] Storelli M., et al., Trace elements in loggerhead turtles (Caretta caretta) from the eastern Mediterranean Sea: overview and evaluation. Environmental pollution, 2005. 135(1): p. 163–170. doi: 10.1016/j.envpol.2004.09.005 15701403

[39] Burger J., et al., Metals and metalloids in tissues of American alligators in three Florida lakes. Archives of Environmental Contamination and Toxicology, 2000. 38(4): p. 501–508. 10787102 10.1007/s002449910066

[40] Burger J., et al., Arsenic, cadmium, chromium, lead, mercury and selenium concentrations in pine snakes (Pituophis melanoleucus) from the New Jersey Pine Barrens. Archives of environmental contamination and toxicology, 2017. 72(4): p. 586–595. doi: 10.1007/s00244-017-0398-5 28424837

[41] Kienzle E., et al., Chemical composition of turtles and tortoises. The Journal of nutrition, 2006. 136(7): p. 2053S–2054S. doi: 10.1093/jn/136.7.2053S 16772495

[42] Clauss M., Tannins in the nutrition of wild animals: a review. Zoo animal nutrition, 2003. 2: p. 53–89.

[43] Helary S.F., et al., Black rhinoceros (Diceros bicornis) natural diets: comparing iron levels across seasons and geographical locations. Journal of Zoo and Wildlife Medicine, 2012. 43(3s). doi: 10.1638/2011-0153.1 23156705

[44] Puls R., Mineral Levels in Animal Health: Diagnostic Data. 2nd ed. ed. 1994, Clearbrook, BC, Canada: Sherpa International.

[45] Ganz T. and Nemeth E., Regulation of iron acquisition and iron distribution in mammals. Biochimica et Biophysica Acta (BBA)-Molecular Cell Research, 2006. 1763(7): p. 690–699. doi: 10.1016/j.bbamcr.2006.03.014 16790283

[46] Fisher G.L., Function and homeostasis of coper and zinc in mammals. Science of the Total Environment, 1975. 4(4): p. 373–412. doi: 10.1016/0048-9697(75)90029-7 1105784

[47] Sereshk Z.H. and Bakhtiari A.R., Concentrations of trace elements in the kidney, liver, muscle, and skin of short sea snake (Lapemis curtus) from the Strait of Hormuz Persian Gulf. Environmental Science and Pollution Research, 2015. 22(20): p. 15781–15787. doi: 10.1007/s11356-015-4631-3 26036580

[48] Dierenfeld E.S., et al., Nutrient composition of prey items consumed by free-ranging Drymarchon couperi (Eastern indigo snakes). Southeastern naturalist, 2015. 14(3): p. 551–560.

